# Hierarchical Classification of Urban ALS Data by Using Geometry and Intensity Information

**DOI:** 10.3390/s19204583

**Published:** 2019-10-21

**Authors:** Xiaoqiang Liu, Yanming Chen, Shuyi Li, Liang Cheng, Manchun Li

**Affiliations:** 1 School of Geography and Ocean Science, Nanjing University, Nanjing 210093, China; 2 Jiangsu Provincial Key Laboratory of Geographic Information Science and Technology, Nanjing University, Nanjing 210093, China; 3 Collaborative Innovation Center for the South Sea Studies, Nanjing University, Nanjing 210093, China

**Keywords:** airborne laser scanning, hierarchical classification, intensity, geometry, supervised learning, unsupervised learning

## Abstract

Airborne laser scanning (ALS) can acquire both geometry and intensity information of geo-objects, which is important in mapping a large-scale three-dimensional (3D) urban environment. However, the intensity information recorded by ALS will be changed due to the flight height and atmospheric attenuation, which decreases the robustness of the trained supervised classifier. This paper proposes a hierarchical classification method by separately using geometry and intensity information of urban ALS data. The method uses supervised learning for stable geometry information and unsupervised learning for fluctuating intensity information. The experiment results show that the proposed method can utilize the intensity information effectively, based on three aspects, as below. (1) The proposed method improves the accuracy of classification result by using intensity. (2) When the ALS data to be classified are acquired under the same conditions as the training data, the performance of the proposed method is as good as the supervised learning method. (3) When the ALS data to be classified are acquired under different conditions from the training data, the performance of the proposed method is better than the supervised learning method. Therefore, the classification model derived from the proposed method can be transferred to other ALS data whose intensity is inconsistent with the training data. Furthermore, the proposed method can contribute to the hierarchical use of some other ALS information, such as multi-spectral information.

## 1. Introduction

By the year 2050, 68% of the world population is expected to live in urban areas [1], thus increasing the importance of urban morphology and ecology research, in which two-dimensional land cover products are commonly used [2,3]. However, two-dimensional land cover products cannot represent the vertical differentiation of geo-objects. Thus, airborne laser scanning (ALS), which can directly acquire three-dimensional geometry information of geo-objects, has been introduced into urban morphology and ecology research [4,5], such as change detection [6,7] and carbon storage mapping [8,9], Unfortunately, a lot of different types of geo-objects, such as buildings, vegetation, and cars, may appear in a small urban local neighborhood [10,11], and it is difficult to automatically extract all geo-objects in the urban environment from raw ALS geometry information. Consequently, many researchers only extracted the ground [12,13], the buildings [14,15], or the powerlines [16,17]. On the other hand, geometry information acquired by ALS was often integrated with passive multi- or hyper-spectral remote sensing image for land cover classification [18,19,20,21]. Nevertheless, the automatic fusion of ALS and passive remote sensing image faces its own challenges, owing to the ALS data and the optical images having different characteristics [22,23,24].

Besides recording points some ALS devices also record spectral information called “intensity”, which can enable the separation of man-made and natural geo-objects [25,26]. With the development of full-waveform and multi-spectral laser scanning, the intensity information will become an important part of the ALS data [4,27]. As compared to the fusion spectral from passive remote sensing image, the intensity recorded by ALS has several advantages: (1) it is independent of illumination conditions such as shading since LiDAR instruments provide their own light source; (2) ALS allows a vertical differential of the intensity; and, (3) spectral mixing within the measured intensity is minimized [4].

Many researchers utilized the supervised learning method with both geometry and intensity information to classify the ALS data [28,29,30]. However, the intensity recorded by ALS is fluctuating due to airborne flight height, atmospheric transmittance, detector responsivity, in-situ calibration, time conditions, and others [31], which resulted in the supervised classifier, directly trained using both geometry and intensity information, not being robust, and being hard to transfer to other ALS data. On the other hand, intensity information is more important to distinguish ground-level geo-objects, such as roads and low vegetation [32,33], and it is not elegant to ignore this once this is available.

This paper proposes a method using supervised learning for the stable geometry information and unsupervised learning for the fluctuating intensity information of the ground-level points to reasonably utilize the intensity information recorded by ALS. The proposed method includes three core parts. First, it uses the training data with the geometry feature to train the supervised classifier, and then the trained supervised classifier is used to classify the ALS data. Second, it extracts ground-level points from the classified ALS data and utilizes unsupervised learning with intensity information to reclassify these ground-level points. Finally, it combines the results of the supervised and unsupervised learning by the heuristic rule.

The main contributions of our proposed method are as follows. (1) In the first part, the geometry feature used in the proposed method contains “absolute feature” (e.g., height, normal) varied depending on transformation and rotation, and “relative feature” (e.g., distribution of points), which is invariant to transformation and rotation. This concatenated geometry feature represents the distribution of point neighborhood and the intuition of geo-objects with special direction and height. (2) In the second part, unsupervised learning does not need to be trained and its parameters can be re-estimated in ALS data to be classified. Thus, the classification model that is derived from the proposed method is more robust to fluctuating intensity. (3) In the third part, a heuristic rule is designed to integrate the urban ALS classification results of the supervised and unsupervised learning, which is based on our defined confidence of supervised and unsupervised learning classification results.

After providing a brief summary of related work in Section 2, we clearly explain the proposed method in Section 3. Subsequently, the results are shown in Section 4 and they are discussed in Section 5. Finally, we provide concluding remarks as well as suggestions for future work in Section 6.

## 2. Related Work

ALS data classification belongs to point clouds classification and can be categorized into point-wise and segment-wise method according to the classification primitive. The point-wise method individually classifies each point of the ALS data by using the respective features as the inputs for a standard supervised classifier [10,34]. The segment-wise method first divides the ALS data into segments and then assigns class labels to the segments so that all points within a segment obtain the same class label [35]. The segment-wise method uses more features, like segment size and shape, and therefore obtains a smoother classification result than the point-wise method. However, the performance of the segment-wise method is negatively affected by under- and over-segmentation errors [35]. On the contrary, the point-wise method directly classifies the ALS data point by point without segmenting and extracting features from the segmented object; hence, its result reveals a “pepper and salt noise” behavior, because it ignores the correlation between the labels of the nearby pixels [11,34,36].

Context information, which is the semantical label of a point similar to its nearby points, is usually introduced to smooth the point-wise classification. Schindler gave an overview and comparison of some commonly used filter methods, such as the majority filter, the Gaussian filter, the bilateral filter, and the edge-aware filter for remote sensing image [36], and these methods can be easily adapted to point clouds. Unlike these filter methods, which only handle local information, in the global smoothing method the entire classification of point clouds uses a graph structure, where the contextual information is contained in the adjacent edge of the graph. Conditional random field (CRF) is a commonly used graph model and it adds pairwise potential alongside the unary potential to model the contextual information. Schindler adopted the Potts model (or the contrast-sensitive Potts model) to calculate the pairwise potential and then inferenced the final classification by the approximating method, such as using the α-expansion graph cut and semiglobal labeling [36]. Niemeyer et al. used the random forest (or linear model) with feature concatenating two neighboring points to learn the pairwise potential [10]. This method of learning the pairwise potential is also the called contextual learning method. The contextual learning method considers the smooth in the training time and obtains more contextual information; for instance, it is more probable that cars are situated on a street than on grassland [10]. Secondly, the contextual learning method based on segments can obtain long-range interactions [30,35]. However, it requires the extensive computation of contextual classifiers and the extraction of useful interaction features. The post-processing smoothing technique, such as global smoothing method in [36], is time efficient and flexible enough to be conducted on point clouds without training an additional supervised classifier as compared to the contextual learning method.

Another post-processing smoothing technique is regularization. Landrieu et al. considered the problem of spatially smoothing semantic classification of point clouds from a structured regularization perspective, whose goal is to find an improved labeling with increased spatial smoothness while remaining as close as possible to the initial classification [34]. Under the framework that was proposed in [34], Li et al. utilized optimal graph and probabilistic label relaxation to handle large wrongly labeled regions [28].

The unary potential of CRF and the regularization method both used initial labeling derived with the standard classification. Hence, this study focuses on the standard classification rather than these smooth methods considering contextual information, because the better its result is, the better further processing will perform [37]. A variety of supervised learning methods have been applied for ALS classification, including support vector machine [38,39], boosting [40,41], and random forest [10,42]. In these classification methods, the generation of good features is a vital part of obtaining a good performance.

There have been three major geometry features: covariance-based features, histogram-based features, and deep features. The covariance-based feature is derived from a three-dimensional covariance matrix of a point and its neighbors [43]. Dittrich et al. investigated the accuracy and robustness of the covariance feature [44]. The histogram-based feature accumulates information regarding the spatial interconnection between a point and its neighborhood into a histogram [45,46,47,48]. Point feature histograms and its improved versions, denoted as fast point feature histograms (FPFH), were typical histogram-based features. The deep feature is learned from training data by using deep neural networks, which have been rapidly growing in recent years. However, the interpretability has been identified as a potential weakness of deep neural networks [49,50]. The covariance feature is usually used in the ALS classification, whereas the histogram-based feature is more rare. Therefore, this study tests the feasibility of the histogram-based feature in large scale ALS point clouds.

Except for the geometry feature, the intensity was usually used to extract some feature [29,30,51], but it is fluctuating, owing to the system and environmental induced distortions. [52,53] improved the classification accuracy of the airborne LiDAR intensity data by calibrating the intensity. A few factors, such as incidence of angle, range calculated with GPS assistant, and atmospheric effect derived from simulating or insite measurement, should be considered while calibrating the intensity, as reasonably utilizing the intensity recorded by ALS is difficult. Apart from these classifications that are needed to calibrate intensity, this study attempts to use unsupervised learning to handle intensity, which does not require calibration of intensity and can be transferred to other ALS data whose acquired condition differs from the training data.

## 3. Materials and Methods

### 3.1. Data Used in This Study

**Figure 1:**
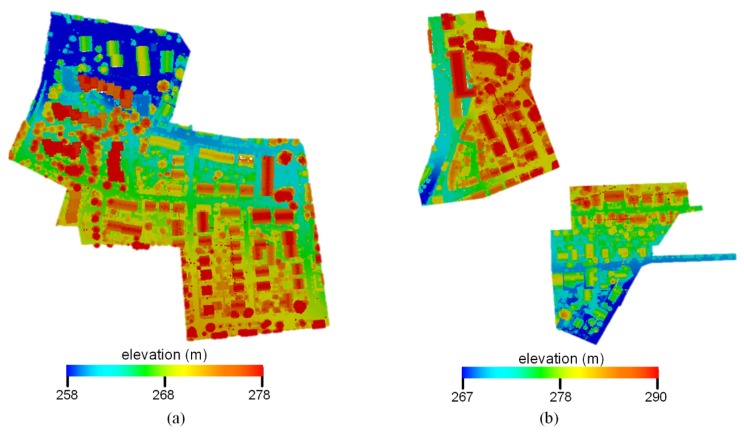
International Society for Photogrammetry and Remote Sensing (ISPRS) benchmark data: (**a**) training data, (**b**) testing data.

This study used the benchmark data (as shown in Figure 1) provided by the International Society for Photogrammetry and Remote Sensing (ISPRS) [54] to evaluate the performance of the proposed method. This ALS data was acquired by Leica Geosystems using a Leica ALS50 system with a 45° field of view and a mean flying height of 500 m above the ground. The average strip overlap was 30%, and the mean point density was eight points **·** m^−2^. Multiple echoes and intensities were recorded. The number of points with multiple echoes was relatively low, owing to the leave-on-condition at the time of the data acquisition. From the scanned data, the ISPRS 3D Semantic Labeling Contest selected two data subsets for the three-dimensional labeling challenge. In total, 9 classes have been defined, namely power line (PL), low vegetation (LV), impervious surface (IS), car (Car), fence/hedge (Fence), roof (Roof), façade (Façade), shrub (Shrub), and tree (Tree). The training and testing areas are from Vaihingen city. The training area consists of a few high-rising buildings surrounded by trees and a purely residential neighborhood with small, detached houses (as shown in Figure 1a). It covers an area of 399 m × 421 m and contains 753,876 points. Dense, complex buildings and some trees characterize the testing area (as shown in Figure 1b), which covers an area of 389 m × 419 m and contains 411,722 points.

### 3.2. Graphical Overview of the Proposed Method

**Figure 2:**
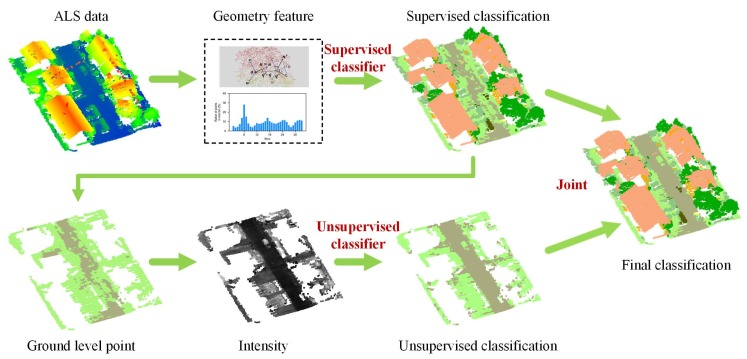
Flowchart of the proposed method. The above row shows the supervised learning for geometry information; the bottom row shows unsupervised learning for intensity information, and the final col joint the supervised and unsupervised classification results.

Figure 2 gives a graphical overview of the proposed method, and it consists of three major components:

- Supervised learning for geometry information: extract geometry feature from the ALS data and then classify the ALS data by using the trained supervised classifier. The geometry feature in this study included FPFH, normal, and height. Four common supervised learning methods, namely decision tree (DT), random forest (RF), support vector classification (SVC), and extreme gradient boost (XGBoost) were used alone to test the performance of the proposed method.
- Unsupervised learning for intensity information: after applying the supervised classifier on the ALS data, the ground-level points and the elevated points (such as building and tree) can be split from the ALS data. The ground-level points were reclassified while using an unsupervised learning method based on intensity information. The Gaussian mixture model (GMM) was the selected unsupervised classifier, because the probability distribution of the intensity of some geo-objects is approximately Gaussian distribution [31].
- Join the classification results of the supervised and unsupervised classifier: for the elevated points, the label was the result of the supervised classifier, whereas, for the ground-level points, the label was the selection from the supervised classification result and the unsupervised classification result based on the heuristic rule. Section 3.3 describes this heuristic rule.

The classifiers derived from the supervised and unsupervised learning should be soft, that is, their outputs should be probabilities for all classes. These probabilities help to construct the heuristic rule.

### 3.3. Supervised Learning for Geometry Information

#### 3.3.1. Geometry Feature Description

The feature in this study was the combination of a relative feature (i.e., FPFH (***fpfh***)), and two absolute features (i.e., normal (***n***) and height (*h*)). Hence, the feature vector was (***fpfh***, ***n***, *h*), in which ***fpfh*** is a 33-dimensional vector, and ***n*** is a three-dimensional vector. FPFH was proposed in [46]. For each point ***q*** of the ALS data, all of ***q***’s neighbors enclosed in the sphere with a given radius *r* are selected (*r*-neighborhood). Given every pair of points, ***q*** and ***q***′, in the *r*-neighborhood, and assuming ***q*** is the point with a smaller angle between its associated normal and the line connecting the point (that is, $n\cdot (q^{\prime }-q)\geq n^{\prime }\cdot (q-q^{\prime })$, a Darboux ***uvw*** frame can be defined while using

(1) $$\{\begin{array}{l} u=n\\ v=\frac{\left(q^{\prime }-q\right)}{{\left\|q^{\prime }-q\right\|}_{2}}\times u\\ w=u\times v \end{array}$$

**Figure 3:**
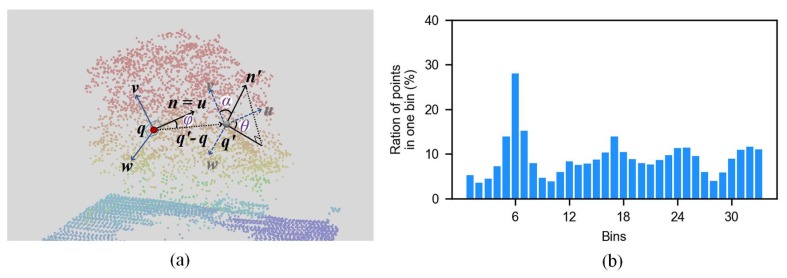
Angular representation (**a**) and an example of Fast point feature histograms (FPFH) (**b**)**.**

Subsequently, the normal $n^{\prime }$ can be represented as an angle tuple (α,θ,φ) in the ***uvw*** frame (as shown in Figure 3a):

(2) $$\{\begin{array}{l} \alpha =v\cdot n^{\prime }\\ \varphi =u\cdot \frac{\left(q^{\prime }-q\right)}{{\left\|q^{\prime }-q\right\|}_{2}}\\ \theta =\arctan\left(w\cdot n^{\prime },\;u\cdot n\right) \end{array}$$

A set of the angle tuples for ***q*** and each point in its *r*-neighborhood can be obtained. Thus, α (θ,ϕ) in the angular tuples can be binned into a histogram 11 bins and then these three histograms are concatenated to form a simple point feature histogram (SPFH). FPFH (as shown in Figure 3b) is the weighted sum of SPFH in the *r*-neighborhood of ***q***.

(3) $$\mathrm{FPFH}\left(q\right)=\mathrm{SPFH}\left(q\right)+\frac{1}{\left|N\left(q\right)\right|}\underset{q^{\prime }\in N\left(q\right)}{\sum }\frac{\mathrm{SPFH}\left(q^{\prime }\right)}{{\left\|q^{\prime }-q\right\|}_{2}}$$

where N(q) represents the neighborhood of ***q*** and |N(q)| represents the number of points in the *r*-neighborhood. More details about FPFH can be found in [55].

FPFH describes the inertial property of the point distribution and is invariant to the transformation and rotation. However, the façade, roof, tree, and other geo-objects have their own direction, and the designed feature for the ALS data classification should represent this fact. Therefore, the point normal was locally estimated by fitting a plane using the neighborhood points [56]. On the other hand, the raw geometry information that was acquired by the ALS system is under the World Geodetic System 1984 coordinate system. It is necessary to obtain the height based on the ground. In this study, a progressive morphological filter that was accomplished in the point cloud library was used to extract the ground due to its good performance and high efficiency in urban environment [57].

#### 3.3.2. A Brief Introduction to Supervised Learning Methods

In this study, four common supervised learning methods, decision tree (DT), random forest (RF), support vector classification (SVC), and extreme gradient boost (XGBoost), are used to process geometry feature. The following is a brief introduction to these methods.

##### Decision Tree

Decision tree predicts the label of a target by learning simple decision rules that are represented using a tree structure. The nodes in DT are split using a best feature and a rational value. Gini index or entropy index is used to calculate the best feature and its divided value. The prediction of an unseen point ${\hat{L}}_{\mathrm{DT}}$ is the fraction of samples of the same class in a leaf that the unseen point falls into:

(4) $${\hat{L}}_{\mathrm{DT}}=\frac{M}{\mathrm{sum}\left(M\right)}$$

where ***M*** is a vector containing the count of samples per class. The predicted class ${\hat{l}}_{\mathrm{DT}}$ is the one with highest probability:

(5) $${\hat{l}}_{\mathrm{DT}}=\underset{c\in L}{\mathrm{argmax}}{\hat{L}}_{DT}\left(c\right)$$

where L is the label space.

##### Support Vector Classification

For binary classification, the data can be separated by a hyperplane:

(6) $$f\left(x\right)=\omega^{T}\phi \left(x\right)+b$$

where ω and *b* are the parameters in a hyperplane, ϕ(x) maps x into a higher-dimensional space. Many hyperplanes might classify the data. SVC aims to find the best hyperplane, so that the distance from it to the nearest data point on each side is maximized, which is written as:

(7) $$\underset{\omega ,b,\zeta }{\min}\;\frac{1}{2}\omega^{T}\omega +C\overset{n}{\underset{i=1}{\sum }}\zeta_{i}\;\mathrm{subject}\;\mathrm{to}\;y_{i}\left(\omega^{T}\phi \left(x_{i}\right)+b\right)\geq 1-\zeta_{i},\;\zeta_{i}\geq 0,\;i=1,\ldots ,\;n$$

where C>0 is regularization parameter, $\zeta_{i}$ is slack variables. This object function can be solved using Lagrange multipliers and sequential minimal optimization [58], and $\omega =\overset{n}{\underset{i=1}{\sum }}y_{i}\alpha_{i}\phi {\left(x_{i}\right)}^{T}$. For an unseen point, the binary label can be derived while using:

(8) $${\hat{l}}_{\mathrm{SVC}}=\mathrm{sgn}\left(\sum_{i=1}^{n}y_{i}\alpha_{i}\phi {\left(x_{i}\right)}^{T}\phi \left(x\right)+b\right)\;\;=\mathrm{sgn}\left(\sum_{i=1}^{n}y_{i}\alpha_{i}\kappa \left(x_{i},\;x\right)+b\right)$$

where κ(·, ·) is a kernel that is introduced because of the dimension of ϕ(x) too high to calculate $\phi {\left(x_{i}\right)}^{T}\phi \left(x\right)$. Radial basis function (RBF) is a popular kernel in remote sensing data processing and it is also used in this study.

SVC can be easily adapted to a multi-class task by using the one-vs-one strategy. In addition, SVC does not provide the probability output. Scikit-learn uses Platt scaling to calibrate the probability of SVC in the binary case [59]. In the multiclass case, probabilities are extended as per [60].

##### Random Forest

Random forest for classification is an ensemble of unpruned classification decision trees [61,62]. Each decision tree in random forest is built from a sample drawn with the bootstrap sample from all of the training data. When splitting a node during the construction of the decision tree, the split that is the best divided among a random subset of the features rather than among all features. The prediction of an unseen point can be the probability vector ${\hat{L}}_{\mathrm{RF}}$ of all classes or a single class ${\hat{l}}_{\mathrm{RF}}$. The predicted probability vector ${\hat{L}}_{\mathrm{RF}}$ of the unseen point is a vote by decision trees ${\hat{L}}_{\mathrm{DT}}$ in the RF, weighted by their probability estimates:

(9) $${\hat{L}}_{\mathrm{RF}}=\frac{1}{n}\overset{n}{\underset{i=1}{\sum }}{\hat{L}}_{\mathrm{DT}i}$$

where, ${\hat{L}}_{\mathrm{DT}i}$ is the probability vector of a decision tree output and *n* is the number of decision trees in the RF. The predicted class ${\hat{l}}_{\mathrm{RF}}$ is the one with highest probability:

(10) $${\hat{l}}_{\mathrm{RF}}=\underset{c\in L}{\mathrm{argmax}}{\hat{L}}_{\mathrm{RF}}\left(c\right)$$

where L is the label space.

##### Extreme Gradient Boost

Extreme gradient boost is tree-boosting ensemble method, its fundamental function predicts a new classification membership after each iteration. XGBoost is built using an additive way that continuously makes new weak classifiers to improve the performance of the previous classifiers. Incorrectly classified samples receive higher weights at the next step, which forcs the classifier to focus on their error in the following iterations [63,64]. The final classification of XGBoost combines the improvement of all the previous modeled trees:

(11) $${\hat{L}}_{\mathrm{XGB}}=\mathrm{softmax}(\overset{n}{\underset{i=1}{\sum }}f_{i}\left(x\right)),\;f_{i}\in F$$

where, f is a function in the functional space and F is the set of all possible DTs. The predicted class ${\hat{l}}_{\mathrm{XGB}}$ is the one with highest probability:

(12) $${\hat{l}}_{\mathrm{XGB}}=\underset{c\in L}{\mathrm{argmax}}{\hat{L}}_{\mathrm{XGB}}\left(c\right)$$

where L is the label space.

### 3.4. Unsupervised Learning for Intensity Information

The GMM is one of the prototype-based clusterings that describe the dataset by a (usually small) set of prototypes [65]. The GMM uses the Gaussian probability model as a prototype to model the dataset and it assumes that the dataset is sampled from the Gaussian mixture distribution

(13) $$p_{M}(x)=\overset{m}{\underset{k=1}{\sum }}\omega_{k}{g\;(x|\mu }_{k},\;\Sigma_{k})$$

where, ***x*** is a *D*-dimensional continuous-value data (i.e., intensity in this study); $\omega_{k}$ are the mixture weights; and, *m* is the number of components. Each component is a Gaussian probability density function with mean vector $\mu_{k}$ and covariance matrix $\Sigma_{k}$

(14) $$g\left({x|\mu }_{k}{,\;\Sigma }_{k}\right)=\frac{1}{{\left(2\pi \right)}^{\frac{D}{2}}{\left|\Sigma_{k}\right|}^{\frac{1}{2}}}e^{-\frac{1}{2}{\left({x-\mu }_{k}\right)}^{T}\Sigma_{k}{}^{-1}\left({x-\mu }_{k}\right)}$$

The mixture weights $\omega_{k}$ satisfy the constraint $\overset{m}{\underset{k=1}{\sum }}\omega_{k}=1$. $\omega_{k}$, $\mu_{k}$, and $\Sigma_{k}$ are the three parameters that need to be estimated. The iterative Expectation-Maximization algorithm or the Maximum A Posteriori estimation is often used to estimate these parameters; more details regarding the estimation method and the implementation can be found in [66] and the Scikit-learn package.

Given each point with intensity ***x***, the output ${\hat{L}}^{\prime }$ of GMM for cluster $\lambda_{k}$ is

(15) $${\hat{L}}^{\prime }{(\lambda }_{k}|x)=\frac{\omega_{k\;}{g(x|\mu }_{k},\;\Sigma_{k})}{\sum_{k=1}^{m}\omega_{k\;}{g(x|\mu }_{k}{,\;\Sigma }_{k})}$$

and the cluster ${\hat{l}}^{\prime }$ is determined by

(16) $${\hat{l}}^{\prime }=\underset{k\in \{1,2,\;\cdots ,m\}}{\mathrm{argmax}}\;{\hat{L}}^{\prime }{(\lambda }_{k}\left|x\right)$$

It is noted that: (1) the smoothing intensity before feeding to GMM made these parameters $\omega_{k}$, $\mu_{k}$, and $\Sigma_{k}$ more reliable; (2) the result of GMM is the cluster, which has no semantic information. The semantic information can be manually set according to the knowledge or automatically determined by the dominant value of the supervised learning result.

### 3.5. Joint Classification Results of the Supervised and Unsupervised Classifier

The outputs of the supervised and unsupervised learning have different meanings. The output of the supervised learning was the probability vector of both ground-level and elevated geo-objects, whereas the output probability vector of the unsupervised learning was only for ground-level geo-objects. Therefore, directly comparing the probability outputs of supervised and unsupervised learning cannot obtain a rational classification result, which is why this study used the ratios $r_{s}$ and $r_{u}$ to represent the confidence of supervised and unsupervised learning classification results.

Without any loss of generality, we assume there were two types of ground-level points, ${\mathrm{glo}}_{1}$ and ${\mathrm{glo}}_{2}$. $r_{s}$ and $r_{u}$ were defined as Equations (17) and (18). In order to make $r_{s}\geq 1,r_{u}\geq 1$, we utilized max and min operation. If $\hat{L}{(\mathrm{glo}}_{1})\geq \hat{L}{(\mathrm{glo}}_{2})$, the probability that the supervised learning classifies an unseen point as ${\mathrm{glo}}_{1}$ was $r_{s}$ times the probability of classifying the unseen point as ${\mathrm{glo}}_{2}$. $r_{u}$ had the same meaning. Thus, we can compare $r_{s}$ and $r_{u}$ to the joint classification results of the supervised and unsupervised classifier. In addition, the joint coefficient (a in Equation (19)) was introduced to represent the tradeoff between the supervised classification result and the unsupervised classification result.

(17) $$r_{s}=\frac{\max\;(\hat{L}{(\mathrm{glo}}_{1}),\hat{L}{(\mathrm{glo}}_{2}))}{\min\;(\hat{L}{(\mathrm{glo}}_{1}),\hat{L}{(\mathrm{glo}}_{2}))}$$

(18) $$r_{u}=\frac{\max\;({\hat{L}}^{\prime }{(\mathrm{glo}}_{1}),{\hat{L}}^{\prime }{(\mathrm{glo}}_{2}))}{\min\;({\hat{L}}^{\prime }{(\mathrm{glo}}_{1}),{\hat{L}}^{\prime }{(\mathrm{glo}}_{2}))}$$

(19) $$y\;=\{\begin{array}{c} \hat{l}\;;\;ar_{s}\geq r_{u}\\ {\hat{l}}^{\prime }\;;\;ar_{s}<r_{u} \end{array}$$

A set of combinations of two types would be obtained if there were three or more types of ground-level points. For every combination, the above rule would be used, and then the selection would be determined by voting. The combined result is smoothed using the majority filter to obtain a more homogeneous classification result.

The proposed method was implemented by using python 3.6 in Jupyter Notebook. The code can be found from: https://github.com/LidarLiu/ALS_Classification_Hierarchically_Geometry_Intensity.

## 4. Results

**Figure 4:**
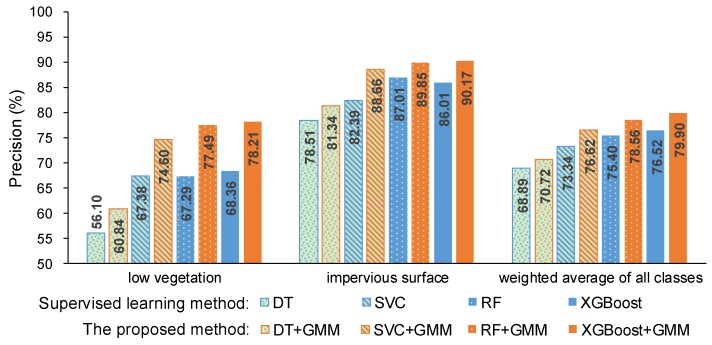
Precision of the supervised learning method without intensity and the proposed method.

**Figure 5:**
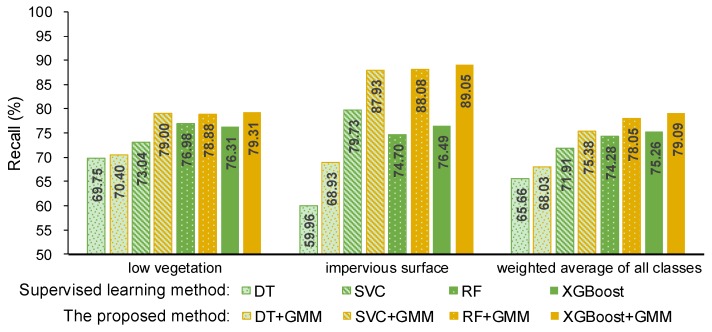
Recall of the supervised learning method without intensity and the proposed method.

**Figure 6:**
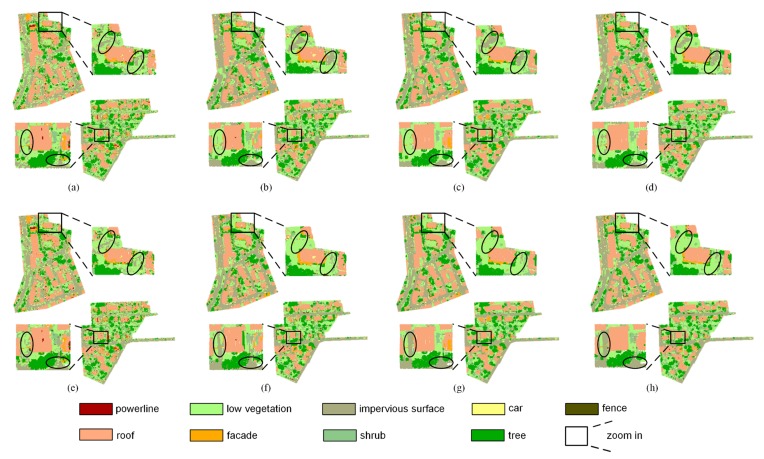
Classification result of testing data. Upper row is the result of supervised learning methods without intensity: (**a**) decision tree (DT), (**b**) support vector classification (SVC), (**c**) random forest (RF), (**d**) extreme gradient boost (XGBoost). Bottom row is the result of the proposed method which joint the result of supervised and unsupervised learning methods: (**e**) DT + Gaussian mixture model (GMM), (**f**) SVC + GMM, (**g**) RF + GMM, and (**h**) XGBoost + GMM.

**Figure 7:**
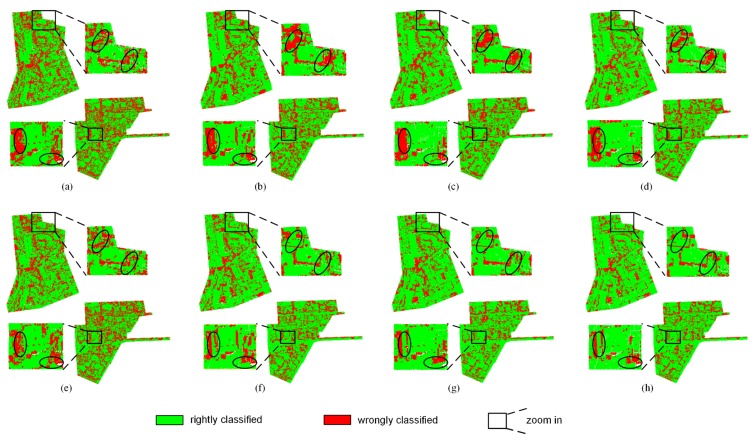
Classification error of testing data. Upper row is the classification error of supervised learning methods without intensity: (**a**) DT, (**b**) SVC, (**c**) RF, (**d**) XGBoost. Bottom row is the classification error of the proposed method: (**e**) DT + GMM, (**f**) SVC + GMM, (**g**) RF + GMM, and (**h**) XGBoost + GMM.

Here, precision and recall were used to measure the performance of the proposed method and they are shown in Figure 4 and Figure 5. The precision and recall of the proposed method are higher than supervised learning method without intensity, especially for the low vegetation and the impervious surface, which can also be found in Figure 6 and Figure 7. A comparison of the bottom row and the upper row in Figure 6 (or Figure 7) depicts that: 1) some misclassified low vegetation points were revised to the impervious surface (as shown in lower left inset); 2) some misclassified impervious surface points were revised to low vegetation (as shown in upper right inset). These two revisions increased the precision and recall of the low vegetation and the impervious surface. For all of the selected supervised learning methods, the precision of low vegetation was greatly increased: DT increased by 4.47%, SVC increased by 7.22%, RF increased by 10.20%, XGBoost increased by 9.85% (as shown in Figure 4); and, the recall of impervious surface was significantly increased: DT increased by 8.97%, SVC increased by 8.2%, RF increased by 13.38%, and XGBoost increased by 12.56% (as shown in Figure 5). These improvements on ground-level points classification also improved the overall performance measured using the weighted average, which reveals that the proposed method can improve the urban ALS classification accuracy by using intensity.

**Figure 8:**
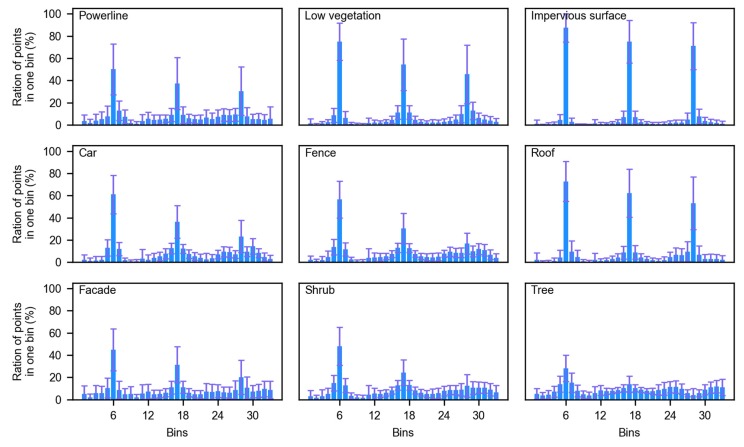
Fast point feature histogram of all geo-objects included in the training data.

Secondly, the fence, the car, and the façade were underestimated (as shown in Table 1), whereas four dominant geo-objects, namely the low vegetation, the impervious surface, the roof, and the tree, had a higher F1 score that is the harmonic mean of precision and recall, which reveals that the imbalance in the environment in the real world influences the classification. The confusion matrix (Table 2) and the visualization of the FPFH of all the geo-objects (Figure 8) were used to analyze the influence. Each geo-object has its own histogram, which helps the supervised learning method to distinguish different geo-objects; however, some geo-object’s FPFH might be confused with others. The powerline is located above the roof and its point density is low, so the powerline points were typically misclassified as the roof. Car points and fence points were mainly misclassified as the shrub, whereas the shrub points misclassified as car or fence were rare, which might be because their height and FPFH are similar and the number of shrub points is much larger than car and fence points (as shown in Table 2). Some roof points and façade points were confused because the edge of the building (i.e., the interaction of roof and façade) is hard to distinguish.

## 5. Discussion

### 5.1. Compare the Proposed Method with the Supervised Learning Method Considering Intensity

#### 5.1.1. Comparison Conditions Setting

One way of utilizing the intensity is to concatenate it with the geometry feature. This study compared the proposed method with the supervised learning method by using the feature (***fpfh***, ***n***, *h*, *i*), in which *i* is the intensity of a point. The intensity information was considered under certain conditions; one condition was the same as that of the training data and another was different from the training data. The ISPRS benchmark testing data is acquired under the same conditions as the training data. The following method was used to simulate the intensity of the testing data under different conditions.

A simplified form of the ALS range equation can be obtained under the assumptions of an extended target (i.e., one that intercepts the entire laser beam) and Lambertian reflectance [31]:

(20) $$P_{r}=\frac{\cos\alpha }{{4R}^{2}}P_{t}D_{r}^{2}\eta_{atm}\eta_{sys}\rho$$

where $P_{r}$ is the received optical power (which is directly proportional to the intensity i), α is the angle of incidence, R is the range between the scanner and the target, $P_{t}$ is the transmitted power, $D_{r}$ is the received aperture diameter, $\eta_{atm}$ is the atmospheric transmission factor, $\eta_{sys}$ is the system transmission factor, and ρ is the target reflectance at the laser wavelength.

The ISPRS benchmark data was acquired at a height of 500 m. This study modified the intensity by simulating its acquisition above a height of 700 m, for which the effect of α on the intensity is negligible when compared to the range. Thus, this study simply modified intensity by using

(21) $$i_{\mathrm{modified}}=\frac{R_{\mathrm{original}}}{\;R_{\mathrm{modified}}}\times i_{\mathrm{original}}\;\approx \;(\frac{500-h}{700-h}{)}^{2}{\times i}_{\mathrm{original}}$$

**Figure 9:**
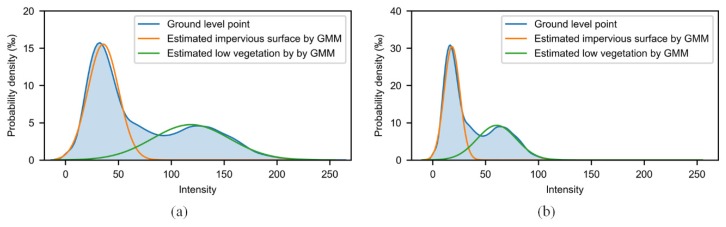
Intensity distribution of ground-level point in the supervised classification result, and GMM result for original intensity (**a**) and modified intensity (**b**).

Another reason to use this simplified equation was that the emission angle of the laser is not recorded in this data. Figure 9 shows the original and modified intensity distribution, in which the result of the GMM is also shown.

The study has also introduced a consistency coefficient to compare the proposed method and supervised learning methods with intensity (i.e., DT_i, SVC_i, RF_i, and XGBoost_i). The consistency coefficient was defined as the ratio of the number of points for which the supervised learning method considering intensity and the proposed method have the same label to the total number of points.

#### 5.1.2. Comparison under the Same Conditions

**Figure 10:**
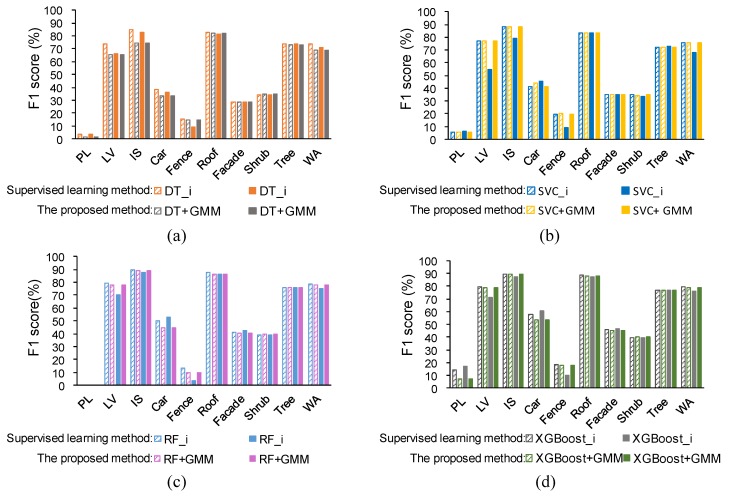
Comparison between supervised learning methods considering intensity and the proposed method under two conditions: same condition was filled with colored slashes, and different condition was filled with solid colors. WA is an abbreviation for weighted average of all classes. The supervised method in (**a**), (**b**), (**c**), (**d**) is DT, SVC, RF and XGboost, respectively.

The comparison result can be seen from Figure 10, where the metrics are the F1 score. The supervised learning methods considering intensity and the proposed method can both obtain better performances than the supervised learning methods without intensity and this improved performance was mainly reflected on the low vegetation and the impervious surface. There was no significant improvement on elevated geo-objects, such as roof, tree, and shrub. This improvement in ground-level geo-objects and the absence of elevated geo-objects indicated that the intensity information is more important for distinguishing ground-level geo-objects.

In addition to DT_i, the performance of the proposed method is similar to the supervised learning method with intensity (as shown in Figure 10). The consistency coefficient between the proposed method and SVC_i, RF_i, XGBoost_i were 91.30%, 93.07%, 94.24%, respectively, which indicates that the classification result of the proposed method is consistent with supervised learning method with intensity. Although DT_i obtained better performance than the proposed method, the performance of DT_i did not exceed RF and XGBoost, which means that DT cannot make full use of the geometry feature and its classification ability is slightly worse. This phenomenon is also shown in Figure 7, where (a) has more red areas than (b), (c), and (d)

#### 5.1.3. Comparison under Different Conditions

Figure 10 also compares the supervised learning methods while considering intensity and the proposed methods under two conditions. It can be concluded that supervised learning method with intensity is sensitive to intensity whereas the proposed method is more robust. For supervised learning method, after modifying the intensity, the F1 score of low vegetation was significantly reduced, which implies that supervised learning, which directly uses the intensity, has some problem. Apart from low vegetation, the impervious surface was slightly decreased because the average intensity of the impervious surface is lower than low vegetation (i.e., the mean of the orange line is smaller than the green line in Figure 9a). The modified intensity was decreased overall. Therefore, the modified intensity of low vegetation is more similar to the original intensity of the impervious surface, which results in a higher recall of the impervious surface and, correspondingly, a higher F1 score.

This study only controlled one factor (i.e., flight height), and some uncertainty appeared in the supervised learning method with intensity. The F1 score of the car significantly increased whereas the F1 score of the fence reduced. Therefore, the intensity of ALS should be carefully used instead of using it directly in supervised learning. In addition, the F1 score of the roof, the tree, the façade, and the shrub were a little varied after modifying intensity, which also evaluated the basic of our method: geometry feature is enough for elevated geo-objects.

### 5.2. The Effect of Joint Parameter on the Performance of the Proposed Method

**Figure 11:**
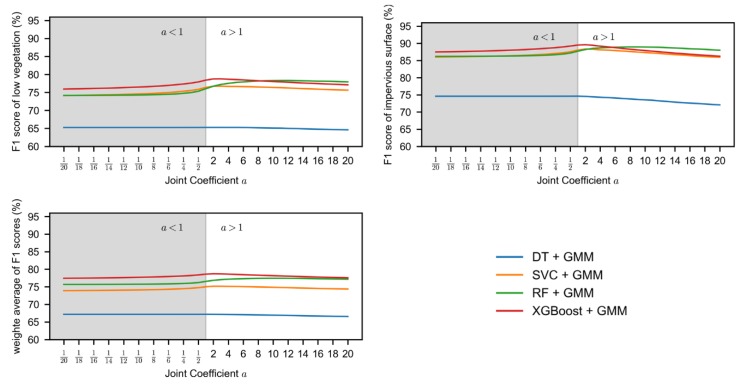
Effect of joint coefficient a on the performance of the proposed method.

The joint coefficient, a, was the key parameter that was used to trade off the result of the supervised learning method and unsupervised learning method. If the joint result was more dependent on unsupervised classification (i.e., *a* < 1 in Figure 11), the performance will decrease. On the contrary, if the supervised classification was considered to be more reliable (i.e., *a* > 1 in Figure 11), the performance would be improved. However, if *a* was very large, it would ignore the result of the unsupervised classification, resulting in the performance decrease. In addition, the value of *a* is changed as the supervised learning methods changes: 1 for DT + GMM, 3 for SVC + GMM, 8 for RF + GMM, and 2 for XGBoost + GMM. Other important parameters can be found in the Appendix A.

### 5.3. The Limitation of the Proposed Method

The classification of façades, fences, and shrubs is relatively poor because of two facts in the ALS data: (1) the geo-objects in the real world are imbalanced (that can be seen from Table 2); (2) the volume density of the fences (0.22 points **·** m^−3^), the façades (0.26 points **·** m^−3^), and the shrubs (1.29 points **·** m^−3^) are lower than the trees (2.26 points **·** m^−3^), the roofs (4.03 points **·** m^−3^), the low vegetations (4.05 points **·** m^−3^), and the impervious surfaces (5.87 points **·** m^−3^). In future work, the rebalance machine learning method should be considered and the optimal or multi-scale radius should be used in the ALS classification, as [43,48]. This study focuses on the intensity information of ALS, so the majority filter is used for small wrongly labeled regions. A graph-structured regularization framework will be considered for large wrongly labeled regions [28,34].

In addition, the type of land cover should be clearly defined to obtain a more generalized ALS classification model and transfer it to other ALS data. This study simply uses the benchmark label. Our further studies will rely on other more complex environments than those found in benchmark data.

## 6. Conclusions

This paper has proposed a novel method that uses the ALS information hierarchically to reasonably utilize fluctuating ALS intensity in a classification method and improve the generalization ability of the classifier. After applying a supervised classifier on the ALS data with the geometry feature, the intensity information is used to reclassify ground-level points by an unsupervised classifier. The final classification result is the integration of the supervised and unsupervised classification results. The proposed method has higher classification accuracy than supervised learning without intensity: in the ISPRS benchmark data, a 5.68 % improvement for low vegetation and a 7.77% improvement for impervious surfaces were achieved.

Unlike supervised learning using intensity, the proposed method handled intensity through unsupervised learning, whose parameters could be re-estimated in the ALS data to be classified, which resulted in the classifier derived from the proposed method being adaptive to the fluctuating intensity and transferrable to other ALS data. Although this study did not prove that a supervised classifier trained both using geometry and intensity information is not useful in principle, it points to the conclusion that one should be cautious about directly using intensity in supervised learning for ALS data classification on a larger scale.

Furthermore, this study utilized the geometry information and intensity information of ALS hierarchically without calibrating intensity, which paves a novel way to use the ALS information and it can exhibit great potential for ALS data processing. However, the type of land cover should be clearly defined, and the imbalance in the real world should be considered in future work to obtain a more generalized classifier and apply it to a three-dimensional urban environment.

## Figures and Tables

**Table 1:** F1 score of the proposed method (%).

Geo-objects→ Method↓	PL	LV	IS	Car	Fence	Roof	Façade	Shrub	Tree	WA
DT + GMM	1.83	65.27	74.62	33.26	14.43	81.84	28.67	34.76	73.23	68.89
SVM + GMM	5.46	76.74	88.29	40.97	19.19	83.15	35.12	34.81	72.16	75.57
RF + GMM	N/A	78.18	88.96	44.76	10.00	86.56	40.18	39.71	76.18	77.80
XGBoost + GMM	7.44	78.76	89.60	53.52	17.93	88.30	45.51	40.10	76.51	79.01

**Table 2:** Confusion matrix between the reference and the predict of the proposed method (RF+GMM as an example). UA is user accuracy and PA is producer accuracy.

Reference→ Predicted↓	PL	LV	IS	Car	Fence	Roof	Façade	Shrub	Tree	Total	UA
PL	0	0	0	0	0	0	0	0	0	0	-
LV	3	77,853	11,558	569	1442	1732	992	5396	929	100,474	77%
IS	0	8811	89,820	417	95	158	244	288	123	99,956	90%
Car	0	17	6	1125	24	13	15	81	38	1319	85%
Fence	0	85	8	342	434	117	5	203	63	1257	35%
Roof	383	3605	218	167	623	90,211	1453	1096	1637	99,393	91%
Façade	84	195	39	5	321	5700	4646	230	678	11,898	39%
Shrub	8	6430	231	1064	3298	2330	1208	10,821	4301	29,691	36%
Tree	122	1694	106	19	1185	8787	2661	6703	46,457	67,734	69%
Total	600	98,690	101,986	3708	7422	10,9048	11,224	24,818	54,226	411,722	
PA	0%	79%	88%	30%	6%	83%	41%	44%	86%		78%

**Figure A1:**
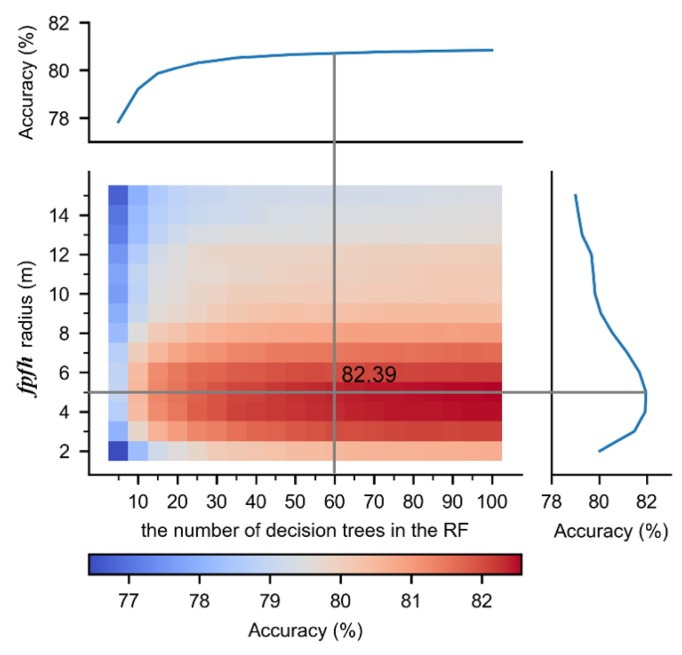
Effect of FPFH radius and the number of decision trees on the accuracy of RF in the proposed method.

**Figure A2:**
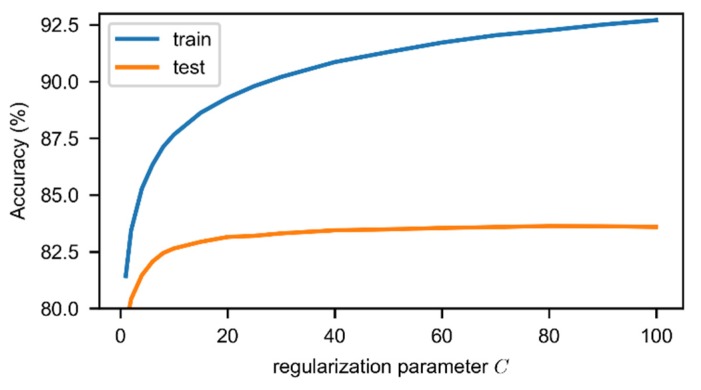
Effect of regularization parameter C on the accuracy of SVC in the proposed method.

**Figure A3:**
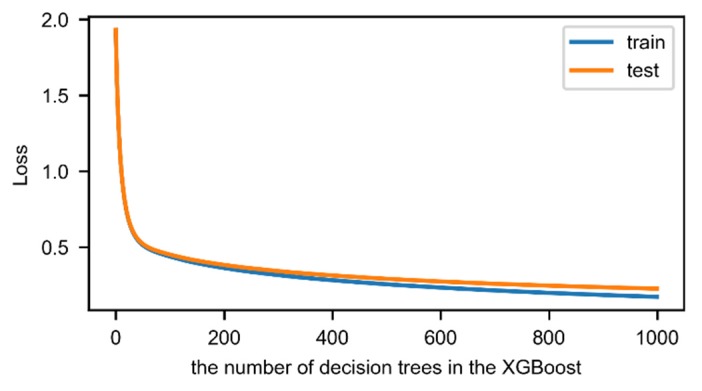
Effect of the number of decision trees on the loss of XGBoost in the proposed method.

**Figure A4:**
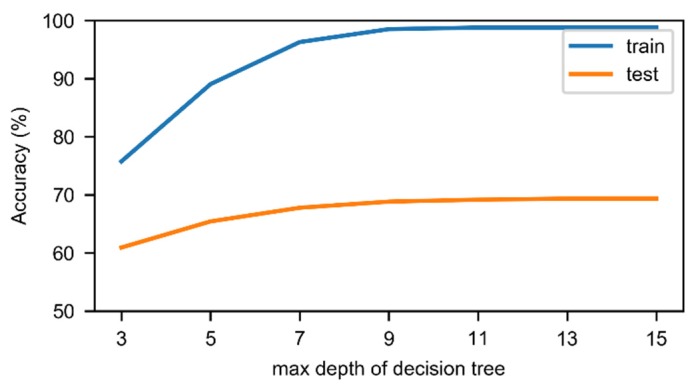
Effect of max depth of decision trees on the accuracy of XGBoost in the proposed method.

## Appendix A

### Appendix A.1. The Parameters in RF and FPFH Radius

We used a 5-fold cross validation method to evaluate the effect of the FPFH radius and the number of decision trees in RF, and the result was shown in Figure A1. The accuracy of RF grows with the number of decision trees and when it exceeds 60, the accuracy of the classifier becomes more stable and cannot significantly improve. Hence, this study set the number of decision trees to 60. On the other hand, the overall accuracy of RF to the FPFH radius is more complex; it first increases and then decreases as the FPFH radius increases. This is caused by the fact that a larger radius contains more geo-objects, so the distinction between different geo-objects becomes smaller. On the other hand, a smaller radius contains fewer points resulting in the neighborhood not containing enough points to represent the geo-object. Hence, this study set the FPFH radius to 5 m.

### Appendix A.2. The Parameter in SVC

We used a 5-fold cross validation method to evaluate the effect of regularization parameter C in the SVC, and the result was shown in Figure A2. The accuracy of SVC grows with the C and when it exceeds 40, the test accuracy of the classifier becomes more stable and cannot significantly improve. Hence, this study set the regularization parameter to 40.

### Appendix A.3. The Parameters in XGBoost

We used a 5-fold cross validation method to evaluate the effect of the number of decision trees and its max depth in the XGBoost, and the result was shown in Figure A3 and Figure A4. The loss of XGBoost decrease with the number of decision trees and when it exceeds 500, the loss of the classifier becomes more stable and cannot significantly reduce. Hence, this study set the number of decision trees to 500. Accordingly, the max depth of decision trees is set to 11.
